# Lack of evidence for condensin or cohesin sequestration on lipid droplets with packing defects

**DOI:** 10.17912/micropub.biology.000497

**Published:** 2021-11-04

**Authors:** Anaïs Mura, María Moriel-Carretero

**Affiliations:** 1 Centre de Recherche en Biologie cellulaire de Montpellier (CRBM), Université de Montpellier, Centre National de la Recherche Scientifique, 34293 Montpellier CEDEX 05, France

## Abstract

Lipid droplets (LD) are organelles born from the endoplasmic reticulum that store fats and sterols in an apolar manner both as an energy reservoir and for protective purposes. The LD is delimited by a phospholipid monolayer covered by a rich proteome that dynamically evolves depending on the nutritional, genetic, pharmacological and environmental cues. Some of these contexts lead to discontinuities in the phospholipid monolayer, termed “packing defects”, that expose LD hydrophobic contents to the surrounding water environment. This triggers the unscheduled binding of proteins with affinity for hydrophobic surfaces, a thermodynamically favorable reaction. We have raised in the past the concern that this titration includes proteins with important roles in the nucleus, which entails a risk of genome instability. Analysis of previously published LD proteomes isolated from cells lacking the transcription factor Ino2p, a prototype of LD bearing packing defects, made us concentrate on two subunits of the cohesin (Smc1p and Smc3p) and one of the condensin (Smc2p) complexes, both essential to promote genome integrity by structuring chromosomes. We report that, in disagreement with the proteomic data, we find no evidence of titration of condensin or cohesin subunits onto LD in *ino2∆* cells. Importantly, during our analysis to label LD, we discovered that the addition of the widely used vital dye AUTODOT^TM^, which emits in the blue range of the spectrum, leads, specifically in *ino2∆,* to the artefactual emission of signals in the green channel. We therefore take the opportunity to warn the community of this undesirable aspect when using this dye.

**Figure 1. Assessment of the co-localization between cohesin or condensin complexes subunits and lipid droplets:**
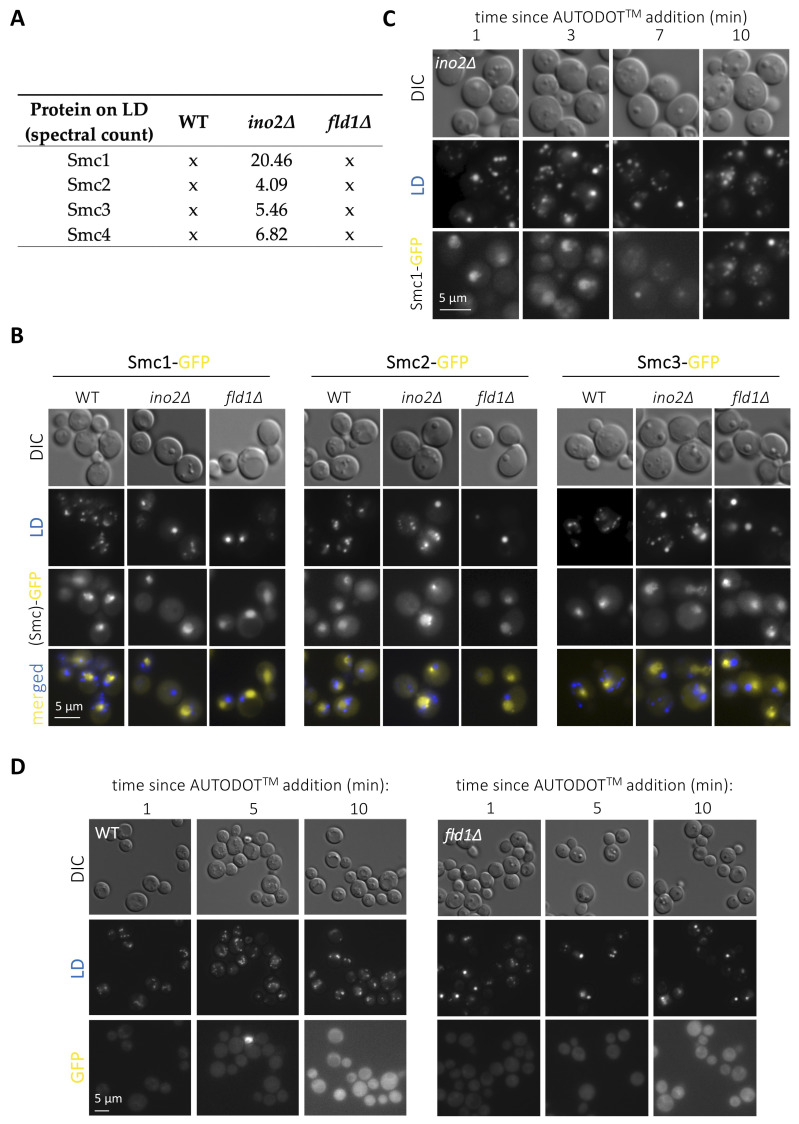
**(A)** Table recapitulating the presence or absence, expressed as spectral counts, of Smc1p, Smc2p, Smc3p or Smc4p in the proteome of lipid droplets (LD) isolated from the indicated wild type (WT) and mutant strains. Data were extracted from (Fei *et al.* 2011b). **(B)** WT, *ino2∆* and *fld1∆* strains were modified to express a C-terminally GFP-tagged version of either Smc1p, Smc2p or Smc3p, grown in complete minimal medium until the exponential phase and imaged to observe GFP signals. To evaluate whether Smc-GFP signals co-localized with LD, the specific dye AUTODOT^TM^, which emits in the blue channel, was added immediately prior to imaging. The shown pictures are representative of the patterns found in all acquired images out of three independent experiments. **(C)** *ino2∆* cells expressing Smc1p-GFP were imaged at the indicated times since AUTODOT^TM^ addition, and both Smc1p-GFP and LD signals monitored. Please note the progressive conversion of Smc1p-GFP-associated signals from nuclear to LD-like. **(D)** WT (left panel) and *fld1∆* (right panel) strains bearing no GFP tagging were imaged at the indicated times since AUTODOT^TM^ addition, and both GFP and blue (LD) channel signals monitored. For all acquisitions, exposure times were 50 ms for AUTODOT^TM^ and 800 ms for GFP.

## Description

Lipid droplets (LD) are organelles that form around neutral lipids that coalesce within the two leaflets of the endoplasmic reticulum (ER) membrane, more frequently the cytoplasmic one, and pop out towards the cytoplasm (Pol *et al.* 2014; Wilfling *et al.* 2014). They are the only organelle of the cell delimited by a monolayer of phospholipids and are present in virtually all species (Beller *et al.* 2010). LD act as an energy reservoir mostly in the shape of triacylglycerols (TAGs) and steryl esters, shelter lipids from unscheduled oxidation and protect the cell from lipotoxicity (Garbarino *et al.* 2009; Bailey *et al.* 2015). Mutations exist that modify the surface and the physico-chemical properties of the LD monolayer. For example, the absence of Cds1p, crucial for phosphatidylcholine synthesis (Klig *et al.* 1988), decreases the availability of phospholipids and, as a consequence, super-sized LD form to spare membrane by increasing the volume-to-surface area ratio (Fei *et al.* 2011a). Further, the monolayers of these LD bear discontinuities, named “packing defects”, that expose their hydrophobic contents to the surrounding water environment. The same defects can occur in mutants lacking Ino2p (Fei *et al.* 2011a), a transcription factor essential for the induction of multiple genes necessary for phospholipid and inositol synthesis (Carman and Henry 2007). Of note, not all mutations giving rise to giant LD are accompanied by packing defects. For example, mutations such as the lack of seipin (Fld1p), which give rise to the severe Berardinelli–Seip lipodystrophy congenital syndrome, support an aberrant flow of TAGs into forming LD, which become super-sized, yet are normally packed (Fei *et al.* 2011a; Wang *et al.* 2014; Wolinski *et al.* 2015). Importantly, the LD surface is covered by a rich proteome under constant evolution (Cermelli *et al.* 2006; Kory *et al.* 2016; Bersuker *et al.* 2018). Part of this proteome supports the function of the LD itself, while the rest constitutes a selective reservoir for other proteins that, this way, can be made available elsewhere in the cell in a regulated manner (Welte 2015).

LD host several proteins fulfilling important roles in nuclear biology. The most prominent and conserved example is histones (Binns *et al.* 2006; Li *et al.* 2012, 2014; Bi *et al.* 2016), whose deposition onto LD regulates their availability during replication thus dictating the rate of nuclear division in *Drosophila* (Li *et al.* 2012, 2014). Some splicing, DNA repair and transcription factors are also regulated at LD (Si *et al.* 2007; Ueno *et al.* 2013; Mejhert *et al.* 2020). Further, we recently demonstrated that a subset of nucleoporins resides on LD, and that the physiological growth or shrinkage of LD during cell growth coordinately sequesters or releases them to adapt nucleo-cytoplasmic transport (Kumanski *et al.* 2021). LD displaying surface packing defects prime the unscheduled binding of proteins with affinity for hydrophobic surfaces, a thermodynamically favorable reaction (Chorlay and Thiam 2020). By using publicly available LD proteomes (Fei *et al.* 2011b), we recently explored the landscape of proteins with nuclear functions reported to be sequestered on LD with packing defects, and found an enrichment in proteins related to chromatin homeostasis and nucleolar biology (Kumanski *et al.* 2021). We are therefore interested in the experimental validation of these hits, as their aberrant binding to mal-packed LD has the potential of titrating them from the nucleus, thus presumably entailing genome instability. We chose to concentrate on subunits of the cohesin (Smc1p and Smc3p) and the condensin (Smc2p and Smc4p) complexes, both essential to promote genome integrity (Yuen and Gerton 2018), yet for which no previous link with LD has ever been reported, to our knowledge. Our re-analysis of the proteomic data reported by (Fei *et al.* 2011b) indicated that the LD purified from *ino2∆* cells attracted all these four subunits specifically, as this was not the case for the WT proteome (Fig. 1A). Further, this was likely to be related to their packing defects, as none of these Smc proteins were retrieved in LD isolated from *fld1∆* cells (Fig. 1A). To validate this experimentally, we grew cells exponentially in complete minimal medium with the goal of simultaneously combining cellular activities requiring LD formation (poor growth medium) with those necessitating cohesin (replication) and condensin (passage through mitosis). We used *Saccharomyces cerevisiae* cells in which either Smc1p, Smc2p or Smc3p had been tagged with GFP at the C-terminal end (Huh *et al.* 2003) and in which LD were stained by the last-minute addition of the specific vital dye AUTODOT^TM^. In WT cells, the three assayed fluorescent Smc proteins yielded a nuclear pattern (Fig. 1B) recapitulative of data published previously when using similarly tagged strains (Bachellier-Bassi *et al.* 2008; Yeh *et al.* 2008; Yahya *et al.* 2020). In agreement with the proteomic data, no signals coming from the cytoplasm or that would co-localize with LD signals could be detected either in WT or in cells bearing super-sized LD but no packing defects, such as *fld1∆* cells (Fig. 1B). Yet, in disagreement with the LD proteome data (Fei *et al.* 2011b), we also failed to detect any cytoplasmic fluorescent signals emitted by the GFP-tagged Smc proteins in *ino2∆* cells (Fig. 1B). Thus, at least under our experimental conditions, the evaluated cohesin and condensin subunits do not seem to be titrated by the LD bearing packing defects of *ino2∆* cells.

Yet, during our analysis, we realized that addition of AUTODOT^TM^ to *ino2∆* cells to visualize LD led to the artefactual emission of signals in the GFP channel (Fig. 1C). This was evidenced by a progressive transformation of the initial Smc1-GFP nuclear signals into cytoplasmic dots until, at 10 minutes, GFP signals fully colocalized with LD ones (Fig. 1C). We add AUTODOT^TM^ at a 20 µM final concentration to our cells immediately prior to mounting and imaging. This procedure allows visualization of LD in the blue channel without major bleed through the GFP one, at least in the 10 minutes-frame needed for image acquisition (Yang *et al.* 2012; Kumanski *et al.* 2021). To reinforce this notion, we evaluated the phenomenon in a WT strain that does not express any GFP fluorophore, and recapitulated minor or no bleed through (Fig. 1D, left panel). It was possible that the smaller size of LD in WT cells makes this artifact less apparent. Yet, *fld1∆* cells, bearing super-sized LD, did not display any major promiscuous GFP signals either (Fig. 1D, right panel). Thus, we warn that the use of AUTODOT^TM^ specifically in *ino2∆* cells, and probably in cells with LD bearing packing defects more generally, leads to artefactual signals in the GFP channel shortly after addition.

Our work highlights the importance of additional experimental validation when considering data obtained from wide proteomic studies. Contrary to our confirmation and characterization of the presence of nucleoporins onto LD (Kumanski *et al.* 2021), we do not confirm the presence of either cohesin nor condensin subunits onto the super-sized LD of cells lacking the transcription factor Ino2p. This argues against further focusing on these factors’ titration as a possible trigger of genome instability in cells bearing LD with packing defects. The study of these potential titration events is relevant because conditions such as obesity are prone to the development of genome instability syndromes like cancer (Deng *et al.* 2016), yet the underlying links are poorly understood. Further, we want to raise awareness that the dye AUTODOT^TM^, known for its violet absorbance and its blue emission in lipophilic environments, and accompanied by a negligible emission in other channels (Yang *et al.* 2012), promiscuously permeates into the green emission channel in *ino2∆* cells. One could imagine that the increased local concentration of apolar molecules as occurring in super-sized LD may trigger a transition justifying this phenomenon, but the same was not observed at the giant LD in *fld1∆* cells. Perhaps the insufficient packing provided by the incomplete monolayer of *ino2∆* LD forces the disorganization of the stored apolar lipids thus altering the emission properties of the intercalated dye molecules.

## Methods

*Saccharomyces cerevisiae* cells were grown at 25°C in complete YNB liquid medium supplemented with 2% glucose. All experiments were performed with exponentially growing cells. For microscopy analyses, 1 mL of the culture of interest was centrifuged; then, the supernatant was thrown away and the pellet was resuspended in the remaining 50 μL. 1 µL of a 1 mM stock AUTODOT^TM^ was added to this volume and, immediately, 3 μL of this cell suspension was directly mounted on a coverslip for imaging at the indicated channels. Fluorescent signals were detected using the adequate wavelength and acquired with a Zeiss Axioimager Z2 microscope and Metamorph software. Subsequent image visualization and analysis were performed with Image J v2.0.0-rc-69/1.52i. The determination of the eventual co-localization of Smc-GFP and LD signals in all cells was done through visual inspection by the experimenter.

## Reagents

The otherwise wild-type strains bearing the GFP-tagged Smc proteins (MM-285, Smc1-GFP; MM-284, Smc2-GFP; MM-283; Smc3-GFP) have been reported in (Huh *et al.* 2003) and were kindly provided by Alenka Čopič, Montpellier. The *ino2∆* and the *fld1∆* deletions were built by classical gene disruption using the G418 resistance cassette *kanMX6* and the hygromycin-resistance cassette *hphMX4*, respectively. We also used AUTODOT^TM ^(SM1000a, Abcepta, San Diego, CA, USA).
